# Night running and internet addiction among university students: a serial mediation model of stress/anxiety and rumination

**DOI:** 10.3389/fpsyg.2026.1776859

**Published:** 2026-06-02

**Authors:** Shaoheng Liang, K. M. Sajjadul Islam, Jiawei Wu, Zitong Ye, Tianyu Gao

**Affiliations:** 1 Guangdong Pharmaceutical University, Guangzhou, China; 2 Department of Computer Science, Marquette University, Milwaukee, WI, United States; 3 Medical College of Wisconsin, Milwaukee, WI, United States; 4 Guangzhou Sport University, Guangzhou, China; 5 School of Physical Education, Jinan University, Guangzhou, China; 6 School of Public Health, Macau University of Science and Technology, Taipa, Macao SAR, China

**Keywords:** internet addiction, night running, rumination, serial mediation model, stress and anxiety

## Abstract

**Background:**

Internet addiction (IA) is prevalent among university students and is often linked to negative affect and maladaptive cognition. Time-specific physical activity such as night running may relate to IA, but the psychological mechanisms remain underexplored.

**Objective:**

This study examined the association between night running and IA and tested whether stress/anxiety and rumination mediate this relationship in a serial pathway.

**Methods:**

A cross-sectional online survey was conducted among 1,138 undergraduates from two universities in Guangdong Province, China. Night running frequency (after 7:00 p.m.) was assessed using an adapted IPAQ-SF item. Stress/anxiety was measured with the DASS-21, rumination with the Ruminative Responses Scale, and IA with the Internet Addiction Test. Correlations were computed and a serial mediation model was tested using bootstrapping.

**Results:**

Night running was negatively correlated with stress/anxiety (*r* = −0.48), rumination (*r* = −0.45), and IA (*r* = −0.52), while stress/anxiety and rumination were positively associated with IA (*r* = 0.42 and *r* = 0.50, respectively). Mediation analyses indicated a significant total effect of night running on IA (*β* = −0.52) and a remaining direct effect (*β* = −0.34). Significant indirect effects were observed via stress/anxiety (*β* = −0.05), via rumination (*β* = −0.07), and via the serial pathway (*β* = −0.06), with all bootstrap 95% CIs excluding zero.

**Conclusion:**

Night running is associated with lower IA among university students, partly through reduced stress/anxiety and reduced rumination, supporting a partial serial mediation model.

## 1 Introduction

Internet addiction (IA)—often operationalized as excessive and uncontrolled internet use accompanied by functional impairment—has become a prominent concern in university populations, where academic demands, flexible schedules, and ubiquitous mobile access can facilitate prolonged online engagement (Kardefelt-Winther, 2014; Romero-López et al., 2021; Kożybska et al., 2022). Recent evidence suggests that IA is highly prevalent among university students worldwide (Romero-López et al., 2021); for example, a 2025 meta-analysis synthesizing 101 studies across 38 countries/territories reported a pooled prevalence of 41.84% and noted a higher risk among males and students with higher depression prevalence in the sampled cohorts (Liu et al., 2025). Given its associations with impaired academic functioning, psychosocial difficulties, and sleep disturbance, clarifying modifiable lifestyle-related correlates and psychological mechanisms of IA in university students remains an important research priority (Tokunaga, 2017; Salari et al., 2025; Wei et al., 2025). University students are an especially relevant population for studying night running. As emerging adults, they generally have greater autonomy over daily routines and leisure-time choices than adolescents, making the post-class evening period a flexible window for social interaction, digital-media use, and physical activity (Pham et al., 2021). College students’ exercise participation is often linked to stress-management and fitness motives, and physical activity has also been associated with better time-management ability in this population (Cao and Jiang, 2025). At the same time, smartphone addiction and bedtime electronic-device use are common concerns among university students and are closely related to sleep and physical-activity behaviors (Zhu et al., 2024). Thus, running after 7:00 p.m. is not merely a general exercise behavior; it is embedded in a university-specific evening context characterized by flexible time use, evening exercise opportunities, and high internet accessibility.

Stress/anxiety is particularly salient in university contexts. Students commonly experience academic and social pressures, and those with elevated stress or anxiety may be especially likely to seek distraction, reassurance, or mood regulation through online activities. In I-PACE terms, stress/anxiety can amplify cue-reactivity and craving for online rewards and reduce self-regulatory capacity, making prolonged internet use more likely (Brand et al., 2016). However, affective states rarely act alone: they are frequently accompanied by repetitive negative thinking styles that prolong and intensify distress. Rumination, a perseverative cognitive process involving recurrent, passive focus on negative feelings and their causes or consequences, is central to response styles theory (Nolen-Hoeksema, 1991) and has been extensively linked to the maintenance and exacerbation of internalizing symptoms (Nolen-Hoeksema et al., 2008). Rumination is also increasingly recognized as relevant to technology-related addictive behaviors: a recent meta-analysis reported that rumination is reliably associated with digital addiction (including problematic internet-related behaviors), supporting the idea that repetitive negative thinking may increase vulnerability to maladaptive online engagement (Gao and Du, 2025).

Rumination may be an especially plausible mechanism linking stress/anxiety to IA for at least two reasons. First, rumination prolongs negative affect and cognitive arousal, reducing opportunities for adaptive problem solving and recovery (Nolen-Hoeksema et al., 2008). Second, rumination is robustly associated with poorer sleep outcomes—such as worse sleep quality and longer sleep onset latency—which can further impair emotion regulation and self-control, thereby increasing susceptibility to excessive internet use as a compensatory strategy. In a systematic review and meta-analysis, higher perseverative cognition (including rumination) showed small-to-medium associations with poorer sleep quality and longer sleep onset latency in non-clinical populations (Clancy et al., 2020). In parallel, IA itself is strongly linked to sleep problems: a meta-analysis in Sleep Medicine Reviews found that individuals with IA had substantially higher odds of sleep problems and shorter sleep duration (Alimoradi et al., 2019). These findings suggest a potentially self-reinforcing loop in which negative affect and rumination disrupt sleep and coping capacity, while excessive internet use further compromises sleep and well-being.

Recent research has also expanded the understanding of how physical activity may relate to internet-related addictive behaviors. Rather than operating only as a general protective factor, physical activity appears to be associated with lower internet addiction through multiple cognitive-affective and self-regulatory mechanisms. In college populations, recent evidence suggests pathways involving rumination and mindfulness, as well as mediation through self-control, indicating that the relationship between physical activity and internet addiction is increasingly being conceptualized in process terms rather than as a simple bivariate association (Peng et al., 2025; Sun et al., 2025). Related evidence further suggests that physical activity may buffer stress-related internet addiction risk among college students, and recent meta-analytic findings indicate that exercise interventions can reduce internet addiction symptoms and related psychological distress in this population (Luo et al., 2024; Yan et al., 2025). Taken together, this emerging literature suggests that contemporary exercise–internet addiction research is moving toward mechanism-based explanations involving affect regulation, repetitive cognition, and self-regulation. This broader evidence base provides further support for the present focus on stress/anxiety and rumination as theoretically relevant mediators in the association between night running and internet addiction.

Within this broader landscape, night running (i.e., running performed in the evening or at night) represents an increasingly common lifestyle pattern among university students, often adopted for stress relief, time management, or fitness goals. In the present study, night running is conceptualized primarily as a potential protective lifestyle behavior in relation to internet addiction (Huang et al., 2024). Compared with general physical activity, night running is theoretically meaningful because it occurs during the evening period, when university students often have greater autonomy over leisure time, higher access to online activities, and increased opportunities for maladaptive internet use. Regular night running may therefore provide an accessible offline coping activity that supports emotional recovery, reduces stress/anxiety, and redirects attention away from problematic online engagement. Importantly, this protective interpretation does not imply that all forms of evening exercise are uniformly beneficial. Physical activity should not be treated as psychologically equivalent across all times of day, because its effects are parameter-sensitive rather than uniform and may vary according to characteristics such as intensity, duration, and timing (Li et al., 2025a). Evening exercise is theoretically distinct because it occurs closer to the sleep period, when physiological arousal, emotional recovery, and pre-sleep cognition are more tightly coupled.

For university students, running after 7:00 p.m. is an ecologically meaningful form of leisure-time exercise because it usually occurs after classes and closer to bedtime. In the present study, post-7:00 p.m. running was used as an operational indicator of night running, not to imply that 7:00 p.m. represents a strict biological threshold, but to capture a time window in which exercise may interact more directly with pre-sleep arousal, emotional recovery, and subsequent cognitive processing. This temporal placement may matter for the proposed affect-cognition cascade. Compared with general physical activity, night running may be more likely to influence internet addiction through two linked pathways: first, by shaping stress/anxiety through affective relief or, when recovery is insufficient, residual arousal and sleep-related strain; and second, by influencing rumination, a maladaptive cognitive style that prolongs distress and may increase reliance on online activities for distraction or mood regulation. Framed in this way, the timing of running is not merely a descriptive feature of behavior, but a theoretically relevant aspect of how exercise may relate to problematic internet use.

Despite the theoretical plausibility of these pathways, several specific gaps remain. First, although previous studies have increasingly examined the association between physical activity and internet addiction, most have treated physical activity as a general protective behavior without distinguishing time-specific exercise, particularly running after 7:00 p.m., from activity performed earlier in the day. This limits understanding of whether evening exercise behaviors have unique relevance for problematic internet use in university students. Second, the psychological mechanisms linking night running to internet addiction remain insufficiently specified. In particular, few studies have examined whether night running is associated with internet addiction through an affect-cognition cascade in which stress/anxiety is linked to rumination and, in turn, to maladaptive internet use. Third, because night running occurs closer to the sleep period, its interpretation should be connected to sleep-related implications such as pre-sleep arousal, bedtime routines, and recovery. However, sleep-related considerations have rarely been integrated into studies of physical activity timing and internet addiction. Addressing these gaps may clarify whether night running functions primarily as a protective behavioral strategy, a potential sleep-related risk factor under certain conditions, or a context-dependent behavior in relation to digital health outcomes.

Therefore, the present study examines the association between night running and internet addiction among university students and tests a serial mediation model in which stress/anxiety and rumination act as potential mechanisms. Consistent with the proposed process framework and the empirical pattern observed in the associations among key variables, we hypothesize that:

*H1:* Night running will be negatively associated with internet addiction;

*H2:* Night running will be negatively associated with stress/anxiety and rumination;

*H3:* Stress/anxiety and rumination will each independently mediate the association between night running and internet addiction;

*H4:* Night running will be indirectly associated with internet addiction through the proposed serial pathway of night running → stress/anxiety → rumination → internet addiction, such that the indirect effect is negative.

## 2 Methods

### 2.1 Research participants

Participants were recruited from two higher education institutions in Guangdong Province, China—Guangdong Pharmaceutical University and Zhuhai College of Science and Technology. Undergraduate students were invited to participate through course instructors at each institution and completed an online questionnaire voluntarily. A total of 1301 questionnaires were returned. After excluding incomplete or invalid responses, 1138 valid questionnaires were retained for final analyses, yielding an effective response rate of 87.5%. The final sample comprised 602 males (52.9%) and 536 females (47.1%). Participants ranged in age from 17 to 25 years (*M* = 20.3, SD = 1.6). With respect to academic year, 298 participants were freshmen (26.2%), 312 were sophomores (27.4%), 281 were juniors (24.7%), and 247 were seniors (21.7%). Regarding institutional distribution, 612 students (53.8%) were from Guangdong Pharmaceutical University, and 526 students (46.2%) were from Zhuhai College of Science and Technology.

### 2.2 Measures methods

#### 2.2.1 Night running

Night running was assessed using an adapted version of the International Physical Activity Questionnaire–Short Form (IPAQ-SF) (Craig et al., 2003), modified to capture time-specific running behavior. Because the original IPAQ-SF does not distinguish physical activity by time of day, a targeted adaptation was implemented to align with the present study’s focus on night-time exercise. Participants were asked: “During the past month, how often did you go running after 7:00 p.m.?” Responses were recorded on a 5-point Likert scale ranging from 1 (never) to 5 (very frequently), with higher scores indicating more frequent engagement in night running. The 7:00 p.m. cutoff was selected to capture a behaviorally meaningful post-class, pre-sleep exercise window in university life and should be interpreted as an operational definition rather than a strict physiological threshold.

A brief self-report frequency item was used because the present study aimed to identify a habitual, time-specific behavioral pattern in a large questionnaire-based sample, rather than to estimate precise energy expenditure, exercise duration, or training load. In this context, a concise frequency-based measure was considered appropriate for reducing participant burden and maintaining feasibility within a large cross-sectional survey. This choice is also broadly consistent with the original purpose of the IPAQ as a practical surveillance-oriented questionnaire rather than a replacement for objective measurement tools (Craig et al., 2003). At the same time, self-reported physical activity measures may show only weak correspondence with accelerometer-based estimates and may be influenced by recall bias, especially when finer-grained features of behavior are of interest. Accordingly, the present measure should be interpreted as an index of perceived frequency of night running rather than a precise behavioral record. Future research should combine self-report with more objective or fine-grained methods, such as wearable-device monitoring, activity logs, or shorter recall protocols, to strengthen validity in time-specific physical activity assessment.

#### 2.2.2 Stress and anxiety

Stress and anxiety were assessed using the Depression Anxiety Stress Scales–21 (DASS-21)(Lovibond and Lovibond, 1995), specifically the stress and anxiety subscales. The stress subscale comprises seven items measuring chronic tension, irritability, and difficulty relaxing, whereas the anxiety subscale includes seven items assessing autonomic arousal and subjective feelings of fear and panic. Participants rated each item on a 4-point Likert scale ranging from 0 (did not apply to me at all) to 3 (applied to me very much or most of the time). In the present study, scores from the stress and anxiety subscales were combined to represent overall levels of stress and anxiety, with higher scores indicating greater psychological distress. The combined scale demonstrated good internal consistency in the current sample (Cronbach’s *α* = 0.88).

#### 2.2.3 Rumination

Rumination was measured using the Ruminative Responses Scale (RRS)(Nolen-Hoeksema and Morrow, 1991). This 22-item scale assesses individuals’ tendency to engage in repetitive and passive thinking about negative emotions and the potential causes and consequences of these emotions. Participants responded to each item on a 4-point Likert scale ranging from 1 (almost never) to 4 (almost always). Total scores were calculated, with higher scores indicating higher levels of ruminative thinking. In the present study, the RRS demonstrated excellent internal consistency (Cronbach’s *α* = 0.91).

#### 2.2.4 Internet addiction

Internet addiction was assessed using the Internet Addiction Test (IAT) (Young, 1998), a widely used self-report instrument designed to measure problematic and compulsive patterns of internet use. The scale consists of 20 items assessing key symptoms such as loss of control, preoccupation, withdrawal, and functional impairment in daily life. Participants rated each item on a 5-point Likert scale ranging from 1 (rarely) to 5 (always). Total scores were computed, with higher scores indicating greater severity of internet addiction. In the present study, the IAT demonstrated high internal consistency (Cronbach’s *α* = 0.93).

### 2.3 Statistics analysis

Data analyses were performed using SPSS version 26.0. First, descriptive statistics were calculated to summarize participant characteristics, and independent sample t-tests and one-way analysis of variance (ANOVA) were conducted to examine differences in study variables by gender and academic year. Second, Harman’s single-factor test was used to assess common method bias, followed by Pearson correlation analysis to explore the associations among night running, stress/anxiety, rumination, and internet addiction. Finally, the PROCESS macro (Model 6) by Hayes was utilized to test the hypothesized serial mediation model, employing a bootstrapping procedure with 5,000 resamples to estimate 95% confidence intervals for the indirect effects.

## 3 Results

### 3.1 Common method bias test

Since the data for all variables (night running, stress/anxiety, rumination, and internet addiction) were collected via self-report questionnaires, common method bias may exist. Procedural remedies, such as anonymous responses and reverse-scored items, were implemented during data collection. Furthermore, Harman’s single-factor test was employed to statistically assess the potential bias before hypothesis testing. All items from the measures of night running, stress/anxiety, rumination, and internet addiction were entered into an unrotated exploratory factor analysis (EFA). The results revealed that 12 factors with eigenvalues greater than 1 emerged, accounting for 68.52% of the total variance. The first factor explained 26.43% of the variance, which is well below the critical threshold of 40%. These findings suggest that common method bias is not a serious concern in the present study.

### 3.2 Descriptive statistical analysis

Gender differences emerged across all key variables (shown in Table 1). Male students reported more frequent night running than female students (*M* = 3.04, SD = 0.93 vs. *M* = 2.78, SD = 0.90), t(1136) = 4.87, *p* < 0.001. In contrast, female students reported higher stress/anxiety (*M* = 15.35, SD = 6.55) than males (*M* = 14.12, SD = 6.20), t(1136) = −3.25, *p* = 0.001, and higher rumination (*M* = 48.50, SD = 10.10) than males (*M* = 46.80, SD = 9.80), t(1136) = −2.88, *p* = 0.004. Male students also showed higher internet addiction scores (*M* = 52.22, SD = 11.80) than female students (*M* = 49.20, SD = 11.30), t(1136) = 4.40, *p* < 0.001. Significant differences were also observed by academic year. Night running frequency decreased across grades, with freshmen reporting the highest levels (*M* = 3.06, SD = 0.90) and seniors the lowest (*M* = 2.72, SD = 0.94), *F*(3, 1,134) = 7.17, *p* < 0.001. In contrast, stress/anxiety increased with academic year, ranging from freshmen (*M* = 13.55, SD = 6.10) to seniors (*M* = 15.90, SD = 6.50), F(3, 1134) = 7.15, *p* < 0.001. A similar upward trend was found for rumination, with mean scores rising from freshmen (*M* = 45.90, SD = 9.60) to seniors (*M* = 49.60, SD = 10.15), F(3, 1,134) = 6.93, *p* < 0.001. Internet addiction also differed significantly by year, showing the lowest levels among freshmen (*M* = 48.20, SD = 11.00) and the highest among seniors (*M* = 53.70, SD = 12.00), F(3, 1,134) = 12.13, *p* < 0.001.

**Table 1:** Descriptive statistics and group differences by gender and academic year for night running, stress/anxiety, rumination, and internet addiction.

Variable	Gender/Grade	*M*	*SD*	Test	*p*
Night running	Male	3.04	0.93	t(1136) = 4.87	<0.001
	Female	2.78	0.90		
	Freshman	3.06	0.90	F(3, 1134) = 7.17	<0.001
	Sophomore	2.99	0.90		
	Junior	2.87	0.92		
	Senior	2.72	0.94		
Stress and anxiety	Male	14.12	6.20	t(1136) = −3.25	0.001
	Female	15.35	6.55		
	Freshman	13.55	6.10	F(3, 1134) = 7.15	<0.001
	Sophomore	14.40	6.20		
	Junior	15.20	6.40		
	Senior	15.90	6.50		
Rumination	Male	46.80	9.80	t(1136) = −2.88	0.004
	Female	48.50	10.10		
	Freshman	45.90	9.60	F(3, 1134) = 6.93	<0.001
	Sophomore	47.10	9.80		
	Junior	48.20	10.00		
	Senior	49.60	10.15		
Internet addiction	Male	52.22	11.80	t(1136) = 4.40	<0.001
	Female	49.20	11.30		
	Freshman	48.20	11.00	F(3, 1134) = 12.13	<0.001
	Sophomore	49.90	11.20		
	Junior	52.00	11.60		
	Senior	53.70	12.00		

### 3.3 Correlation analysis

Descriptive statistics indicated that participants reported a moderate level of night running (*M* = 2.92, SD = 0.92), shown in Table 2. Mean levels of stress and anxiety (*M* = 14.70, SD = 6.35), rumination (*M* = 47.60, SD = 9.95), and internet addiction (*M* = 50.80, SD = 11.60) showed sufficient dispersion, suggesting that the sample exhibited meaningful individual differences across all study variables. Pearson correlation analyses revealed a clear pattern of associations among the variables. Night running was significantly and negatively correlated with stress and anxiety (*r* = −0.48, *p* < 0.01), rumination (*r* = −0.45, *p* < 0.01), and internet addiction (*r* = −0.52, *p* < 0.01). In contrast, stress and anxiety were positively associated with both rumination (*r* = 0.55, *p* < 0.01) and internet addiction (*r* = 0.42, *p* < 0.01). Rumination was also positively correlated with internet addiction (*r* = 0.50, *p* < 0.01). Together, these findings suggest that higher psychological distress and repetitive negative thinking are linked to greater internet addiction, whereas more frequent night running is associated with lower levels of distress, rumination, and problematic internet use, providing initial empirical support for the hypothesized relationships.

**Table 2:** Descriptive statistics and intercorrelations among study variables.

Variable	*M*	*SD*	Night running	Stress and anxiety	Rumination	Internet addiction
Night running	2.92	0.92	—			
Stress and anxiety	14.70	6.35	−0.48**	—		
Rumination	47.60	9.95	−0.45**	0.55**	—	
Internet addiction	50.80	11.60	−0.52**	0.42**	0.50**	—

*p* < 0.05; **p < 0.01.

### 3.4 Testing the mediating effect

As shown in Table 3, night running had a significant total effect on internet addiction [*β* = −0.52, SE = 0.03, 95% CI (−0.57, −0.47)], indicating that higher levels of night running were associated with lower levels of internet addiction. After including stress and anxiety and rumination as mediators, the direct effect of night running on internet addiction remained significant [*β* = −0.34, SE = 0.03, 95% CI (−0.39, −0.29)], accounting for 65.4% of the total effect. In addition, all indirect effects were statistically significant. The indirect pathway through stress and anxiety alone was significant [*β* = −0.05, SE = 0.02, 95% CI (−0.08, −0.02)], accounting for 9.6% of the total effect, while the indirect pathway through rumination alone was also significant [*β* = −0.07, SE = 0.01, 95% CI (−0.09, −0.05)], accounting for 13.5%. Importantly, the serial indirect effect from night running to internet addiction via stress and anxiety and subsequently rumination was significant [*β* = −0.06, SE = 0.01, 95% CI (−0.08, −0.04)], explaining 11.5% of the total effect, indicating that both mediators jointly contributed to the association.

**Table 3:** Mediation effect path analysis.

Type of effect	Path	β	Boot SE	Bootstrap 95% CI		Path effect proportion
				Lower limit	Upper limit	
Total effect	Night running → internet addiction	−0.52	0.03	−0.57	−0.47	100%
Direct effect	Night running → internet addiction	−0.34	0.03	−0.39	−0.29	65.4%
Specific indirect 1	Night running → stress and anxiety → internet addiction	−0.05	0.02	−0.08	−0.02	9.6%
Specific indirect 2	Night running → rumination → internet addiction	−0.07	0.01	−0.09	−0.05	13.5%
Specific indirect 3 (serial)	Night running → stress and anxiety → rumination → internet addiction	−0.06	0.01	−0.08	−0.04	11.5%

**Figure 1:**
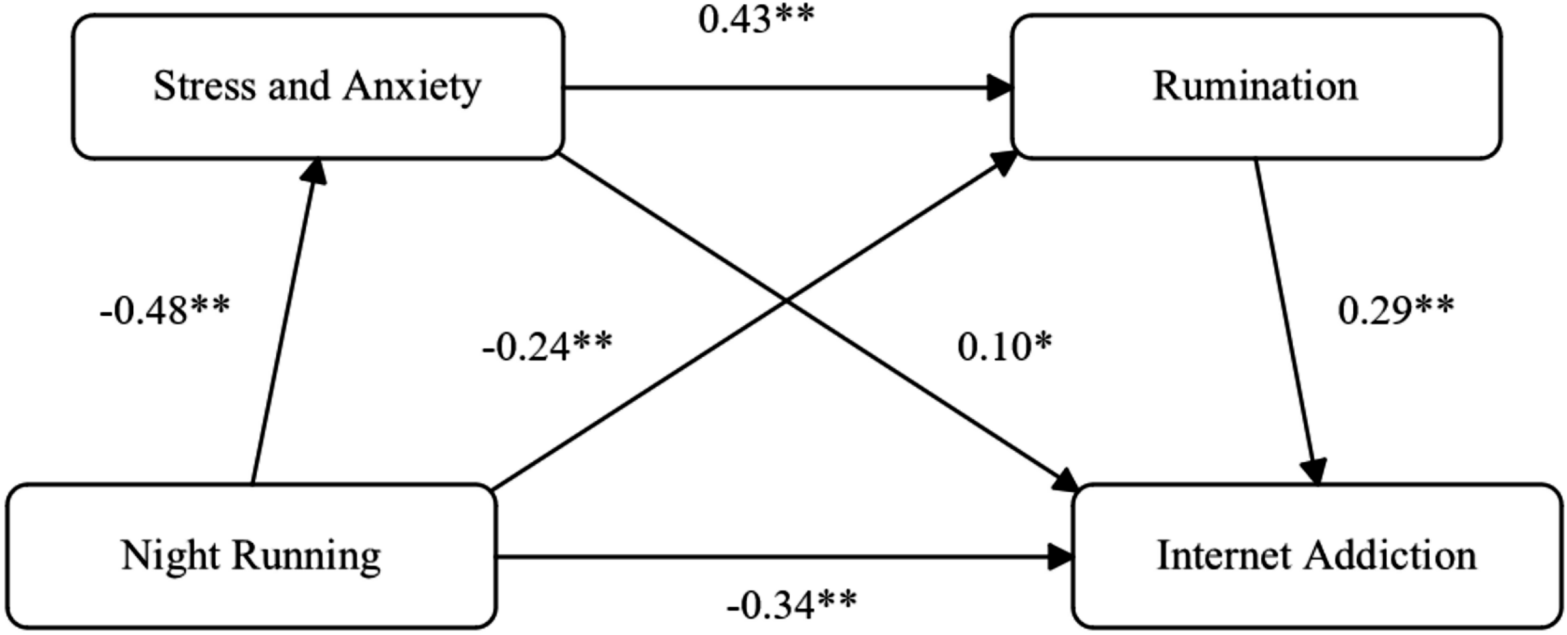
Serial mediation model linking night running to internet addiction via stress and anxiety and rumination.

Figure 1 presents the standardized path coefficients of the serial mediation model. Night running was negatively associated with stress and anxiety (*β* = −0.48, *p* < 0.01), rumination (*β* = −0.24, *p* < 0.01), and internet addiction (*β* = −0.34, *p* < 0.01). Stress and anxiety showed a positive association with rumination (*β* = 0.43, *p* < 0.01) and a smaller but significant direct association with internet addiction (*β* = 0.10, *p* < 0.05). Rumination, in turn, was positively associated with internet addiction (*β* = 0.29, *p* < 0.01). Further comparison of the pathway estimates provides additional insight into the relative importance of the model components. The direct pathway from night running to internet addiction remained the largest component of the total effect, accounting for 65.4%, indicating that the association was only partially mediated by stress/anxiety and rumination. Among the indirect pathways, the rumination-only pathway accounted for the largest proportion of the total effect (13.5%), followed by the serial pathway through stress/anxiety and rumination (11.5%) and the stress/anxiety-only pathway (9.6%). At the path-coefficient level, stress/anxiety showed a stronger association with rumination than with internet addiction, whereas rumination showed a stronger direct association with internet addiction than stress/anxiety did. This pattern suggests that rumination may function as a more proximal cognitive mediator, while stress/anxiety may contribute partly by increasing ruminative thinking. Taken together, the results support a partial serial mediation model in which night running is associated with lower internet addiction both directly and indirectly through reduced stress/anxiety and rumination.

## 4 Discussion

The present study examined the association between night running and IA among university students and tested a serial mediation model involving stress/anxiety and rumination. Overall, the findings supported the proposed model and indicated that night running was associated with lower IA both directly and indirectly through psychological pathways. Correlation results showed that night running was negatively related to stress/anxiety, rumination, and IA, whereas stress/anxiety and rumination were positively related to IA. These associations provided an empirical basis for the mediation tests and align with the study’s hypotheses.

Mediation analyses further clarified how night running may relate to IA risk. Night running demonstrated a significant total effect on IA, and this association remained significant after including stress/anxiety and rumination, suggesting that night running may influence IA through multiple mechanisms. Importantly, both mediators showed significant indirect effects, and the serial pathway (night running → stress/anxiety → rumination → IA) was also significant, indicating that affective relief and reduced perseverative cognition jointly contributed to lower IA. The standardized path model was consistent with this interpretation: night running was negatively associated with stress/anxiety, rumination, and IA, while stress/anxiety positively predicted rumination and (more modestly) IA, and rumination positively predicted IA. The relative magnitudes of the pathways further deepen the interpretation of the model. The direct association between night running and internet addiction accounted for the largest proportion of the total effect, suggesting that stress/anxiety and rumination explained only part of the association. Among the indirect effects, the rumination-only pathway contributed more than the stress/anxiety-only pathway, and rumination showed a stronger direct association with internet addiction than stress/anxiety did. This suggests that rumination may be a more proximal cognitive mechanism linking night running and internet addiction, whereas stress/anxiety may exert part of its influence through increasing repetitive negative thinking. Therefore, interventions that combine physical activity promotion with strategies to reduce rumination may be particularly relevant for reducing problematic internet use among university students.

These findings can be interpreted in light of contemporary process models of problematic internet use. Your introduction highlights the I-PACE framework and compensatory internet use theory, which converge on the idea that negative affective states increase motivation to use the internet for short-term relief and thereby elevate IA risk. In this context, night running may function as a behavioral coping strategy that reduces distress and improves affect regulation, which in turn lowers reliance on online activities for emotion-focused compensation. In addition, the observed link between stress/anxiety and rumination is consistent with the premise that affective dysregulation is often accompanied by repetitive negative thinking that maintains distress and narrows coping options. The current results suggest that night running may be associated with reduced exposure to this “affect–cognition” cascade, which is theoretically central to the development and maintenance of IA.

A more specific explanation for these cognitive shifts may be derived from neurobiobehavioral models of exercise and brain health. Recent neuroimaging meta-analytic evidence suggests that physical exercise improves general cognitive task performance and is associated with increased task-related activation, particularly in the bilateral precuneus, with these neural effects being shaped by factors such as exercise intensity and adherence (Li et al., 2025b). Although this evidence does not address rumination directly, it provides neurobiological plausibility for the idea that exercise may strengthen cognitive operations relevant to disengaging from repetitive negative thinking. In addition, recent review evidence indicates that physical-activity-based interventions can reduce repetitive negative thinking, especially rumination and worry, with stronger effects observed when exercise is sustained over time and combined with psychological training (Wang et al., 2025). One plausible interpretation, therefore, is that night running may not only reduce distress at an affective level, but may also facilitate attentional reallocation away from self-focused negative loops and toward more externally oriented, task-relevant processing. In this way, exercise may weaken the cognitive perseveration that prolongs stress/anxiety and increases reliance on the internet for compensatory regulation.

The independent mediating roles of stress/anxiety and rumination are also meaningful. The indirect effect through stress/anxiety indicates that night running may relate to lower IA partly by alleviating psychological distress, which is consistent with models emphasizing affective drivers of excessive internet use. The indirect effect through rumination suggests that night running may also relate to less maladaptive cognitive perseveration, thereby decreasing the tendency to use online activities as distraction or avoidance. Notably, the serial indirect effect highlights a coherent process: reduced stress/anxiety is associated with reduced rumination, which in turn is associated with lower IA. This pattern fits a response-styles perspective in which rumination prolongs negative affect, undermines effective problem solving, and increases vulnerability to maladaptive coping behaviors.

At the same time, the direct effect of night running on internet addiction remained significant after stress/anxiety and rumination were included, accounting for a substantial proportion of the total effect. This pattern suggests that the present model captures only part of the underlying process and that additional mediating mechanisms are likely involved. Two plausible candidates are sleep-related processes and social support. First, because night running occurs close to the sleep period, its association with internet addiction may partly depend on sleep hygiene, including bedtime regularity, pre-sleep arousal management, and behaviors that either support or disrupt sleep-related recovery. Better sleep hygiene may reduce late-night internet use opportunities and improve next-day self-regulation, whereas poor sleep-related routines may weaken these benefits. Second, night running may also operate through social pathways. For some students, running is not only an individual activity but also a socially embedded behavior that can increase perceived support, companionship, and accountability, which may in turn reduce reliance on online environments for connection, distraction, or mood regulation. Accordingly, the remaining direct effect should not be interpreted as weakening the current model, but rather as indicating that the relationship between night running and internet addiction is likely multifactorial and only partially explained by stress/anxiety and rumination in the present study.

The group differences observed in this study also offer applied insight. Male students reported more frequent night running and higher IA scores, whereas female students reported higher stress/anxiety and rumination. In addition, night running decreased across academic years, while stress/anxiety, rumination, and IA increased, with seniors showing the highest distress-related profiles and IA levels. This pattern may reflect accumulating academic and career pressures as students progress through university, alongside reduced time or motivation for regular exercise. These findings suggest that prevention efforts may benefit from being tailored by subgroup: for example, strengthening adaptive stress-management and rumination-reduction strategies may be particularly relevant for students reporting elevated distress, while interventions for high-risk IA groups may incorporate structured physical activity routines as part of a broader behavioral plan.

Several practical implications follow. First, campus health programs could consider promoting running and other accessible physical activities as a component of digital well-being initiatives, emphasizing their potential role in reducing distress and repetitive negative thinking that are linked to IA. Second, because “night running” encompasses heterogeneous patterns, guidance should be behaviorally specific—encouraging students to avoid very late, highly arousing exercise sessions when these interfere with sleep, and to pair exercise with recovery strategies (e.g., cooldown routines) to reduce cognitive arousal. Third, given the role of rumination, combining physical activity promotion with brief cognitive-behavioral or mindfulness-based components aimed at reducing repetitive negative thinking may enhance impact on IA risk.

These findings should also be interpreted in light of important limitations related to both the dual-edged nature of evening exercise and the contextual specificity of the sample. Although more frequent night running was associated with lower internet addiction in the present sample, evening exercise is not uniformly beneficial under all conditions. As noted earlier, later timing and higher exercise strain may interfere with subsequent sleep and recovery, particularly when exercise occurs close to habitual sleep onset. This complexity is important because sleep disturbance is itself closely linked to problematic internet use and may represent a competing pathway through which very late or highly intense night running could increase, rather than reduce, vulnerability to internet addiction. In addition, because the sample was drawn from only two universities in Guangdong Province, the observed associations may also reflect contextual features of university life in South China. Regional academic stress patterns, employment-related pressure, climate, and local exercise routines may shape both internet-use behavior and the everyday meaning of night running, which may limit the direct generalizability of the findings to other university environments or cultural settings. Accordingly, the protective interpretation of the present findings should be understood as conditional rather than absolute: night running may be beneficial in relation to internet addiction, but its effects likely depend on when it occurs, how intense it is, whether it supports or disrupts subsequent sleep, and the broader context in which the behavior takes place.

Future research can address these limitations by using longitudinal or experimental designs, incorporating objective activity and sleep measures (e.g., wearable devices), and distinguishing exercise timing (e.g., early evening vs. late night), duration, and intensity. In addition, because night running in the present study was assessed using a single self-reported frequency item, future work should employ finer-grained behavioral assessment approaches, such as activity logs, ecological momentary recording, or shorter recall protocols, to reduce recall bias and improve validity in time-specific physical activity measurement.

## 5 Conclusion

In conclusion, this study provides evidence that more frequent night running is associated with lower internet addiction among university students, and this association operates through both affective and cognitive pathways. Night running was negatively correlated with stress/anxiety, rumination, and internet addiction, while stress/anxiety and rumination were positively associated with internet addiction. Mediation analyses further showed significant indirect effects via stress/anxiety, via rumination, and via the proposed serial pathway (night running → stress/anxiety → rumination → internet addiction), alongside a remaining direct effect, supporting a partial serial mediation model. These findings highlight night running as a potentially relevant lifestyle correlate of digital health, suggesting that reducing stress/anxiety and rumination may be key psychological mechanisms linking physical activity timing to problematic internet use.

## Data Availability

The raw data supporting the conclusions of this article will be made available by the authors, without undue reservation.
